# Supplementary material for: Association between oral microbiome and five types of respiratory infections: a two-sample Mendelian randomization study in east Asian population
Source: Front Microbiol. 2024 Apr 10;15:1392473. doi: 10.3389/fmicb.2024.1392473 (PMC11039966; doi:10.3389/fmicb.2024.1392473)
Supplement: Supplementary file 1 [file Data_Sheet_1.zip › Supplementary Materials/MR plots of saliva/Tonsillitis/s__Porphyromonas_sp000467855_mgs_2773/scatter.pdf]

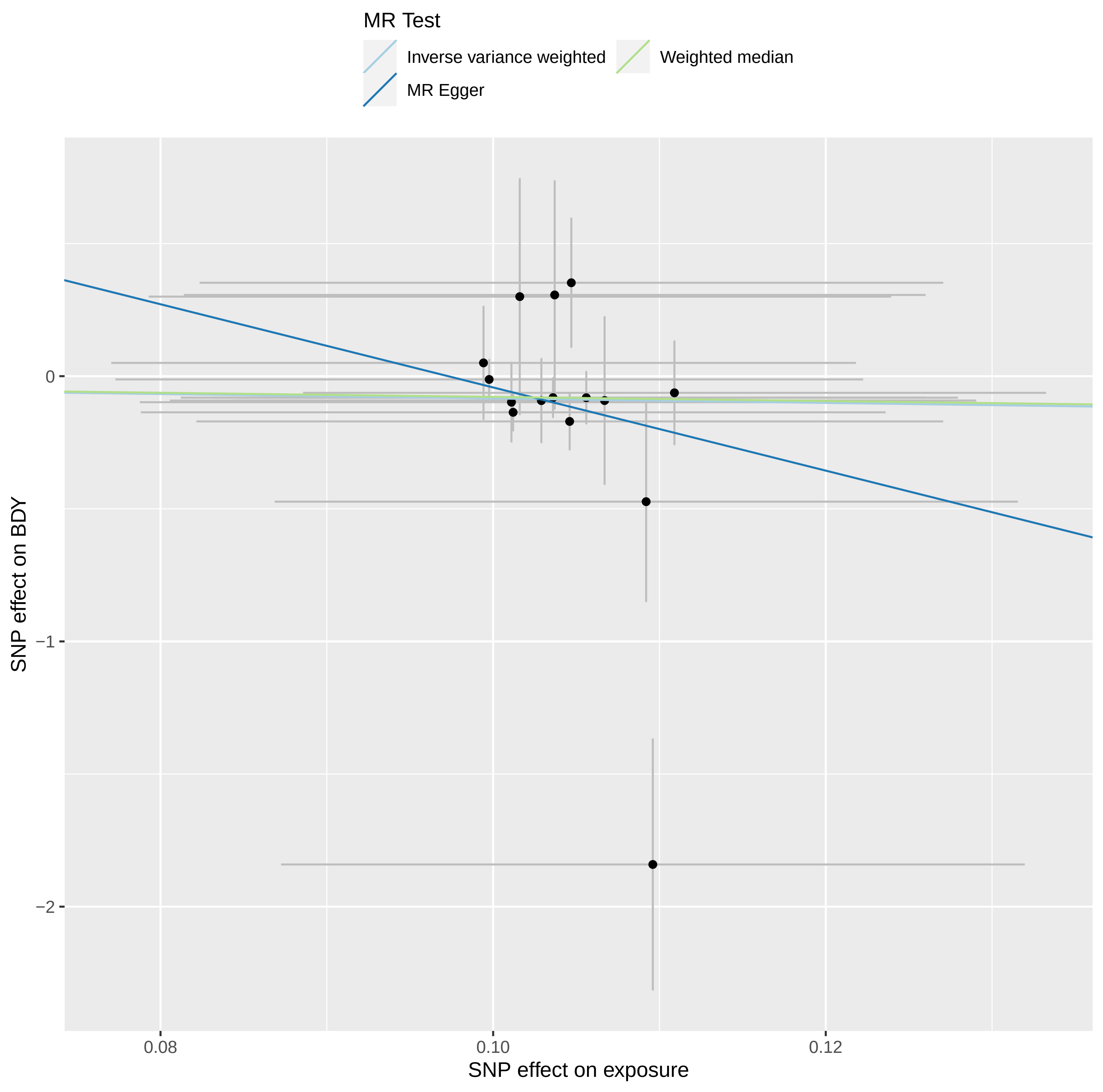

MR Test
Inverse variance weighted
Weighted median
MR Egger
SNP effect on BDY
0
−1
−2
0.08
0.10
0.12
SNP effect on exposure
